# Comparison Study of MS-HRM and Pyrosequencing Techniques for Quantification of *APC* and *CDKN2A* Gene Methylation

**DOI:** 10.1371/journal.pone.0052501

**Published:** 2013-01-11

**Authors:** Francesca Migheli, Andrea Stoccoro, Fabio Coppedè, Wan Adnan Wan Omar, Alessandra Failli, Rita Consolini, Massimo Seccia, Roberto Spisni, Paolo Miccoli, John C. Mathers, Lucia Migliore

**Affiliations:** 1 Department of Translational Research and New Technologies in Medicine and Surgery, Division of Medical Genetics, University of Pisa, Pisa, Italy; 2 Department of Laboratory Medicine, Pisa University Hospital (AOUP), Pisa, Italy; 3 Advance Medical and Dental Institute, University Sains Malaysia, Penang, Malaysia; 4 Department of Clinical and Experimental Medicine, University of Pisa, Pisa, Italy; 5 Department of Surgery, Medical, Molecular, and Critical Area Pathology, University of Pisa, Pisa, Italy; 6 Human Nutrition Research Centre, Institute for Ageing & Health, Biomedical Research Building, Campus for Ageing & Vitality, Newcastle University, Newcastle upon Tyne, United Kingdom; 7 Istituto Toscano Tumori (ITT), Florence, Italy; University of Navarra, Spain

## Abstract

There is increasing interest in the development of cost-effective techniques for the quantification of DNA methylation biomarkers. We analyzed 90 samples of surgically resected colorectal cancer tissues for *APC* and *CDKN2A* promoter methylation using methylation sensitive-high resolution melting (MS-HRM) and pyrosequencing. MS-HRM is a less expensive technique compared with pyrosequencing but is usually more limited because it gives a range of methylation estimates rather than a single value. Here, we developed a method for deriving single estimates, rather than a range, of methylation using MS-HRM and compared the values obtained in this way with those obtained using the gold standard quantitative method of pyrosequencing. We derived an interpolation curve using standards of known methylated/unmethylated ratio (0%, 12.5%, 25%, 50%, 75%, and 100% of methylation) to obtain the best estimate of the extent of methylation for each of our samples. We observed similar profiles of methylation and a high correlation coefficient between the two techniques. Overall, our new approach allows MS-HRM to be used as a quantitative assay which provides results which are comparable with those obtained by pyrosequencing.

## Introduction

Epigenetic processes, including DNA methylation, histone tail modifications and nucleosome positioning, as well as non coding RNAs (ncRNAs), play an important role in modulating gene expression and, therefore, in determining the phenotype [1]–[3]. The most widely studied epigenetic mark is DNA methylation, the covalent addition of a methyl group (CH3), by means of DNA-methyltransferases (DNMTs), to the 5′ position in the nucleotide cytosine principally when this occurs as a CpG dinucleotide. Epigenetic aberrations involving tumor suppressor gene inactivation, oncogene activation, and chromosomal instability play an important role in tumorigenesis [1], [4]. A wide range of environmental exposures and dietary factors can influence epigenetic marks and molecules [5] with important implications for risk of common complex diseases including cancer. Several genes involved in tumorigenesis (e.g. *APC2* and *SFRP4*) are promising epigenetic CRC biomarkers because they are methylated in a large proportion of patients. Moreover aberrant methylation may be detected non-invasively in blood or fecal samples (e.g. *ESR1*, *SEPT9* and *VIM*) as a diagnostic tool [6]–[10]. Hypermethylation of other genes such as *CDH13* and *FLBN3* is associated with poor prognosis in CRC [11] and some pharmacological compounds such as DNMT and histone deacetylase (HDAC) inhibitors are being tested in metastatic CRC patients to improve their survival or quality of life [12]. Altogether these observations highlight the importance of having rapid, cost effective and reproducible methods for quantification of DNA methylation. Many DNA methylation assays are based on PCR reactions after sodium-bisulfite treatment [13]. The gold standard technique for DNA methylation detection is pyrosequencing that is a sequence by synthesis method that analyze bisulfite-modified and PCR-amplified DNA, providing also information on the methylation status of single CpG sites [14]. The differences between methylated and unmethylated DNA after sodium bisulfite treatment can be also evaluated by means of methylation sensitive high resolution melting (MS-HRM) that analyses the melting curves immediately after PCR in a closed-tube system [15]. The relative simplicity, high reproducibility and low cost of MS-HRM makes this technique a good method of choice for methylation assessment in research and diagnostic applications [13], [15]. In the present study we developed a method for deriving single estimates, rather than a range, of methylation using MS-HRM and compared these estimates with those obtained by pyrosequencing. In this context we analyzed the levels of methylation of two CRC-related genes in DNA extracted from CRC tissues by means of MS-HRM. We investigated the tumour suppressor adenomatous polyposis coli gene (*APC*), which encodes a key protein in the WNT signaling pathway and the cyclin-dependent kinase inhibitor 2A gene (*CDKN2A/p16*) that arrests the cell cycle in G1 and G2 phases.

## Materials and Methods

### Tissue samples

DNA was obtained from surgically resected tumor tissues and from adjacent normal tissues (20 cm from the tumour). The study was approved by the ethical committee of the Pisa University Hospital and is sponsored by Istituto Toscano Tumori (ITT) and by the Northumberland Local Research Ethics Committee (project reference NLREC2/2001). The CRC patients gave written informed consent. The 90 biological samples were obtained from the Department of Surgery of the University of Pisa, Italy and from Wansbeck General Hospital, Northumberland, UK.

### Extraction of genomic DNA

Genomic DNA was extracted using QIAmp DNA blood Mini Kit (Qiagen, Milan, Italy) according to the manufacturer's instruction. The extracted DNA was quantified using a Nano Drop ND 2000c spectrophotometer (NanoDrop Thermo scientific, Wilmington, DE).

### Bisulfite modification

200 ng of DNA from each sample were treated with sodium bisulfite using the “EpiTect® Bisulfite Kit” (Qiagen, Milan, Italy) according to the manufacturer's protocol. Sodium bisulfite treatment converts all unmethylated cytosines into uracil, whilst methylated cytosines are left unchanged.

### MS-HRM

**Figure 1:**
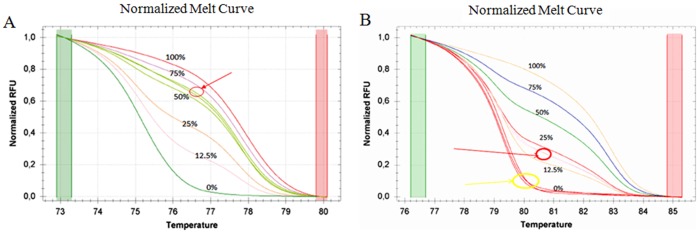
Melting curves of *APC* gene(A): the standards and a sample in duplicate (highlighted); Melting curves of *CDKN2A* gene (B): the standards and two samples in duplicate (highlighted).

For the MS-HRM of *APC* and *CDKN2A* genes we used methylation independent (MIP) primers, based on Huang et al. and Wodjacz et al. [16]–[18] and then developed in-house protocols for the PCR and HRM conditions. All analyses were run according to the following conditions: 1 cycle of 95°C for 12 min, 60 cycles of 95°C for 30 s, Ta for 30 s and 72°C for 15 s; followed by an HRM step of 95°C for 10 s and 50°C for 1 min, 65°C for 15 s, and continuous acquisition to 95°C at one acquisition per 0.2°C. PCR was performed in a final volume of 25 µl, containing 12,5 µl of master mix (Qiagen), 10 pmol of each primer and 1 µl (almost 10 ng) of bisulfite modified DNA template. Each reaction was performed in triplicate. We analyzed 10% of the samples independently on separate occasions to verify the inter-assay variability and we observed a good reproducibility. Figure 1 shows the melting profiles of the two promoter regions analyzed. Table 1 shows the conditions (primers, annealing temperature, CpG sites, and amplicon length) used for each gene. Fully methylated and unmethylated DNA (EpiTect® methylated and unmethylated human control DNA, bisulfite converted, Qiagen, Milan, Italy) were mixed to obtain the following ratios of methylation: 0%, 12,5%, 25%, 50%, 75%, 100%. Standard curves with known methylation ratios were included in each assay and were used to deduce the methylation ratio of each tumor and normal sample. MS-HRM experiments were performed in the Medical Genetics laboratory, University of Pisa.

**Table 1:** MS-HRM and pyrosequencing conditions and analyzed sequences.

Gene	Primer sequences: 5′-3′	Ta	CpG sites	Amplicon lenght	Amplified region (accession and nucleotide numbers)
*APC MS-HRM*	**F**: CGGGGTTTTGTGTTTTATTG; **R**: TCCAACGAATTACACAACTAC	56°C	4	71 bp	NC_00005 (112073406–112073477)
*CDKN2A MS-HRM*	**F**: CGGAGGAAGAAAGAGGAGGGGT; **R**: CGCTACCTACTCTCCCCCTCT	62°C	7	93 bp	NC_000005.9 (c21974935–21974843)
*APC pyro*	**F**: TATTAATTTTTTTGTTTGTTGGGGA; **R**: AACTACACCAATACAACCACATATC; Sequencing primer: GGGGTTTTGTGTTTTATTG	55°C	7	66 bp	NC_00005 (112073426–112073491)
*CDKN2A pyro*	**F**: AGAGGATTTGAGGGATAG; **R**: AATTCCCCTACAAACTTC; Sequencing primer: GGGTTGGTTGGTTATTA	50°C	10	65 bp	NC_000005.9 (c21974900–21974836)

### Pyrosequencing

**Figure 2:**
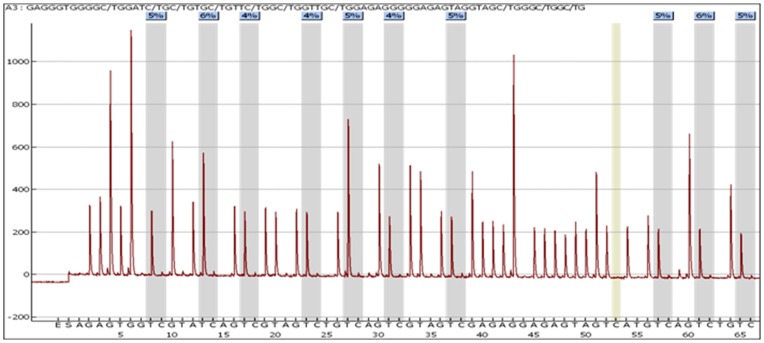
*CDKN2A* pyrosequencing. 10 CpG sites analyzed in the CpG Island of the *CDKN2A* gene. The y axis represents the signal intensity, while the x axis shows the dispensation order. The blue color indicate the % of methylation at each CpG site.

Genomatix software (www.genomatix.de) Gene2Promoter was used to identify the promoter regions of interest and PSQ software was used to design the corresponding primer sets. Bisulfite modified (BM) 0% and 100% methylated DNA were diluted to produce DNA mixtures with defined methylation content (0%, 25%, 50%, 75%, 100%) which were used subsequently for PCR and pyrosequencing. We validated the *APC* and *CDKN2A* pyrosequencing with a pre-PCR standard dilution obtaining good linear correlations (R^2^ = 0.98 and 0.95 respectively) between expected and observed methylation. The standard conditions for the PyroPCR were: 12,5 µL Taq Mastermix (Qiagen), 10 pmol of each primer and 1 ul (almost 25 ng) of BM DNA in a total volume of 25 µL. The PyroPCR temperature profile was the following: 95°C for 15 min, 94°C for 15 s, T_a_ for 30 s, 72°C for 30 s (Repeat steps 2, 3, 4×50 times) and 72°C for 10 min. Table 1 shows the pyrosequencing conditions (primers, annealing temperature and CpG site analyzed) for both *APC* and *CDKN2A*. For the purpose of this study, the mean methylation across all CpG sites analysed was calculated for each sample for each gene and used for comparison with MS-HRM. Figure 2 shows *CDKN2A* pyrosequencing of a CRC sample. Pyrosequencing was performed at the Human Nutrition Research Centre, Institute for Ageing & Health, Newcastle University.

### Derivation of single estimates of methylation using MS-HRM

**Figure 3:**
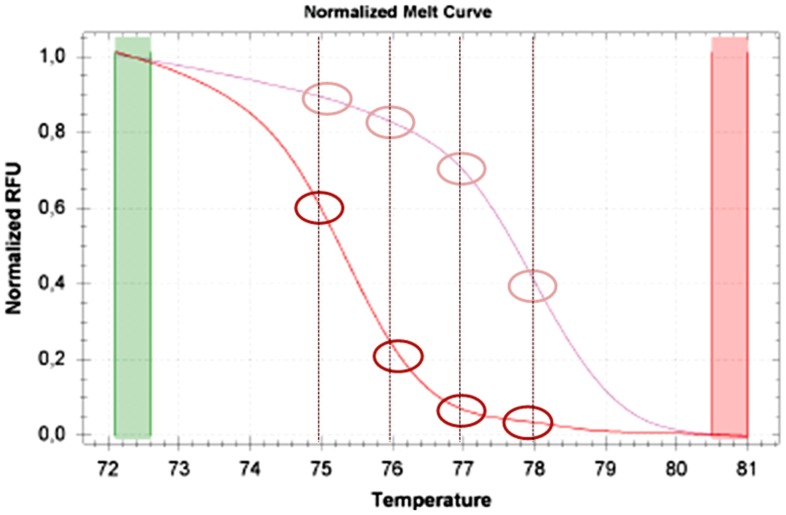
Chosen temperatures to obtain the average of RFU values of the melting curve of each sample. For simplification in the figure we show only the standard curves relative to 0% DNA methylation (lower curve) and 100% DNA methylation (upper curve).

We developed a novel method to derive single methylation percentage values from MS-HRM assays. For this purpose, we used the graphs of normalized melting curves. Figure 3 illustrates the melting curve of the DNA standards and the different fluorescence points, indicated in RFU (Relative Fluorescence Units), showing the melting status of the template at a range of temperatures. In each experiment we derived six RFU values corresponding to DNA standard curves (0%, 12.5%, 25%, 50%, 75%, 100%); each RFU value was estimated as the average of fluorescence units of the temperatures relative to the melting status (from 74°C to 80°C for *APC* gene and from 76°C to 84°C for *CDKN2A* gene: 35 and 45 measures respectively). Starting from this data set, we derived an interpolation curve by the method of interpolating polynomials. For this we used the “polyfit” interpolating function within program MatLab (The MathWorks, Inc., USA) which provides results similar to those obtainable using Lagrange interpolation. Having obtained the interpolation curve of the standards in an experiment, imputation of the observed fluorescence for each sample yields a precise percentage of methylation of the template of interest.

### Statistical analysis

We performed statistical analysis using MedCalc software; we calculated the correlation coefficient between MS-HRM and pyrosequencing techniques for *APC* and *CDKN2A* promoter methylation. Statistical significance was accepted for *P*<0.05. We examined the concordance between estimates of methylation by the 2 methods using Bland-Altman analysis. We used the conventional limits of agreement of Bland-Altman analysis i.e. ±1.96 STD (average difference ±1.96 standard deviation of the difference).

## Results

**Figure 4:**
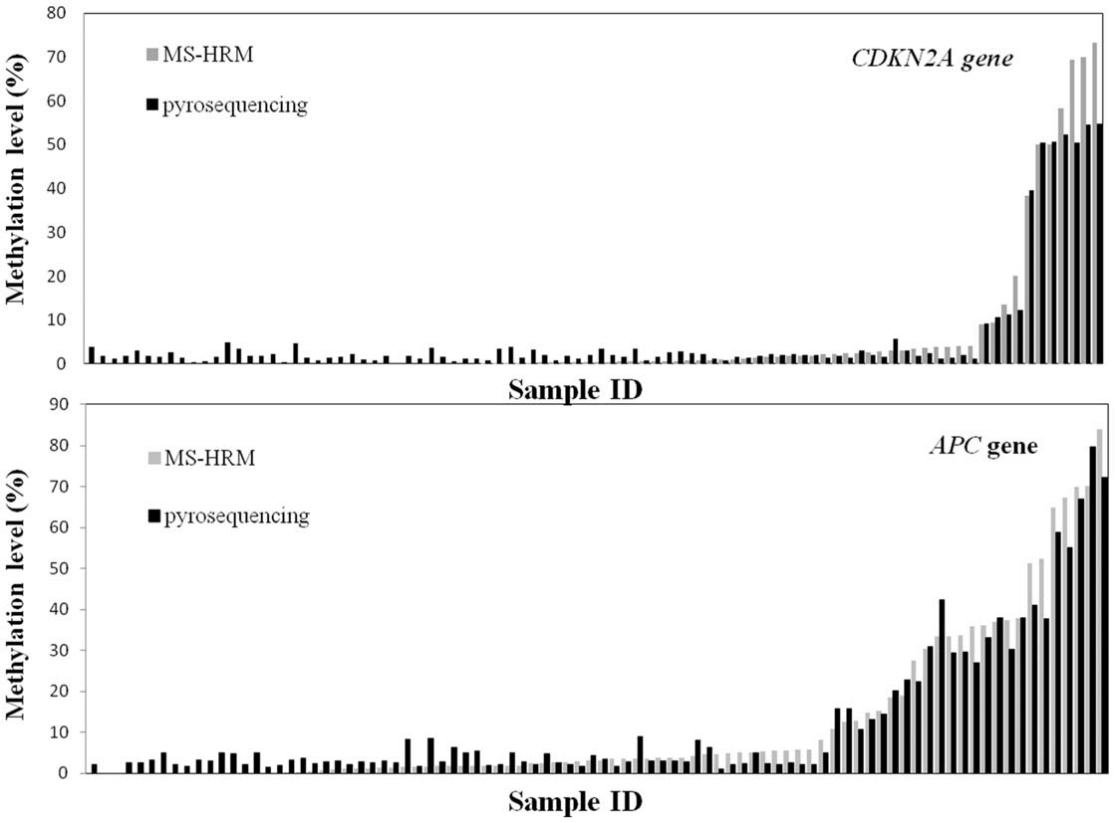
Profile of *CDKN2A* and *APC* methylation (%) obtained for each sample with the two techniques.

**Figure 5:**
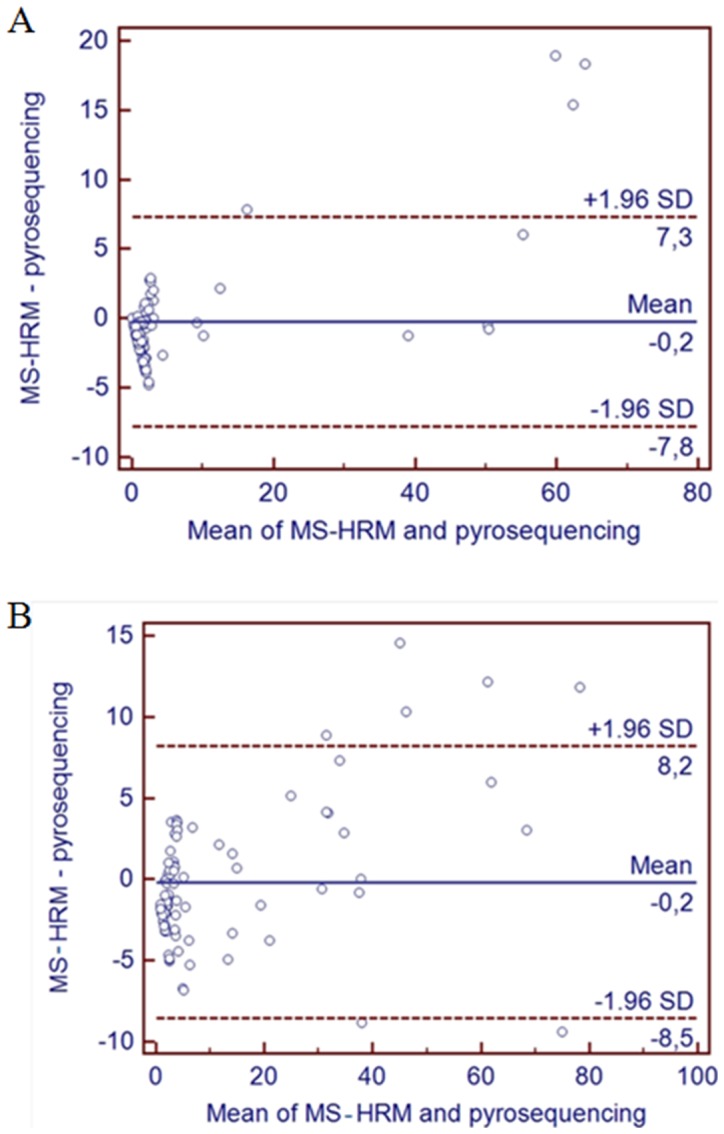
Bland-Altman plots. A) *CDKN2A* gene methylation; B) *APC* gene methylation. MS-HRM and pyrosequencing assays are performed on each sample, resulting in 2*n* data points. Each of the *n* samples is then represented on the graph by assigning the mean of the two measurements as the abscissa (x-axis) value, and the difference between the two values as the ordinate (y-axis) value.

For comparison of the methylation estimates between the two techniques, it was necessary to obtain a precise methylation value by MS-HRM. At each temperature, the reported RFU value within the melt curve, was exported to Microsoft Excel. The RFU average of each DNA standard was then used to obtain an interpolation curve. Finally, imputation of the RFU value (corresponding to the analyzed sample) to the polynomial function provided a precise (single value) estimate of the percentage of methylation. These methylation estimates obtained by MS-HRM assay were then compared with average methylation value derived from pyrosequencing for all the CpG sites within the assay. By using both tumour and normal colorectal tissue we obtained a wide range of DNA methylation levels using the two different techniques (MS-HRM and Pyrosequencing). Figure 4 illustrates the estimates of methylation for *CDKN2A* (7 CpG sites in common analyzed by both methods) and *APC* (4 common CpG sites analyzed) obtained for each sample with the two methods. Correlation coefficients between both methods were high (r = 0,98, P<0.0001 for both genes) indicating that the two techniques detected similar patterns of methylation (Table 2). Using the Bland-Altman plots we observed that, for *CDKN2A*, all estimates of methylation fell within the limits of agreement (mean difference plus and minus 1.96 times the standard deviation of the differences (±1.96 STD) except for 3 samples with relatively high methylation. For these 3 samples, MS-HRM gave a higher percent of methylation than did pyrosequencing (Figure 5A). Repeat analysis by MS-HRM confirmed the relatively high methylation values for these 3 samples. For *APC*, estimation of methylation by the 2 methods fell within the limits of agreement for all samples apart from 5 samples where, again, MS-HRM resulted in higher values of methylation, and one with a lower percent of methylation than that obtained by pyrosequencing (Figure 5B). In general MS-HRM tended to yield lower estimates of methylation with less methylated samples and higher estimates of methylation with more heavily methylated samples than were obtained by pyrosequencing, although the overall mean methylation was very similar for both approaches.

**Table 2:** Coefficient of correlation between MS-HRM and pyrosequencing methylation results.

Parameters	*CDKN2A* gene	*APC* gene
Variable Y	MS-HRM	MS-HRM
Variable X	pyrosequencing	pyrosequencing
Sample size	90	88
Correlation coefficient r	0,9837	0,9787
Significance level	P<0,0001	P<0,0001
95% Confidence interval for r	0,9753 to 0,9893	0,9675 to 0,9860

## Discussion

This investigation demonstrated that our novel approach to quantification of methylation using MS-HRM produced estimates of methylation which were very similar to those obtained by the gold standard pyrosequencing. Across the 90 samples analysed with levels of methylation from zero to >80%, there were no significant differences between the methylation levels obtained by means of the two techniques for both the *APC* and the *CDKN2A* genes. The region within the *APC* gene promoter analyzed by pyrosequencing contained 7 CpG sites, whereas that analysed by MS-HRM included 4 CpG sites. To provide a direct comparison between techniques, we compared the precise percentage of methylation of the 4 CpG sites interrogated in common by both techniques i.e. methylation estimated by the interpolation curve in MS-HRM and the mean % methylation obtained by pyrosequencing. Using a Bland-Altman plot, we demonstrated that these two methods gave similar results despite the use of the two different sets of primers and the correlation coefficient between the two techniques was strong (r = 0,98, P<0.0001). For the *CDKN2A* gene, pyrosequencing provided quantitative estimates of methylation for 10 CpG sites and MS-HRM for 7 CpG dinucleotides. As for the *APC* gene, we compared methylation estimates produced by MS-HRM and by pyrosequencing for the 7 CpG sites in common within the *CDKN2A* gene and we obtained a similar methylation profile with both techniques. There was a high correlation coefficient (r =  0,98, P<0.0001) between techniques but there were a small number of samples (3 out of 90 samples) for which MS-HRM gave higher values than those obtained by pyrosequencing. This apparent bias occurred in samples with greater % methylation. This result might be partially explained by the fact that the MS-HRM primers designed according to Wodjacz et al. [17], [18] include some CpG sites which favor the amplification of a methylated template with respect to an unmethylated one. In contrast, pyrosequencing primers were designed for regions that do not contain CpG sites. Alternatively, heterogeneous methylation across the CpG sites could influence the melting profile of the PCR product and, therefore, the estimates of methylation obtained by the MS-HRM technique (see below for further discussion). Some random errors or bias in individual samples could have arisen through procedures for DNA sample processing and bisulfite conversion since MS-HRM and pyrosequencing were carried out in two different laboratories.

Very recently, Newman et al. attempted to utilize MS-HRM technology to obtain quantitative estimates of DNA methylation for the repetitive sequence LINE 1 elements. The authors defined a value, called Net Temperature Shift (NTS), as the integral difference in the melt curves of a given sample compared with that of the methylated control sample, providing quantitative measurement of differences in methylation. In other words the subtraction of the methylated control normalized curve from each test normalized curve was performed and the summed difference of the fluorescence value at each temperature point (0.1°C intervals) within the entire melt range (10°C) was divided by 100 to obtain the average distance between the curves, or the NTS [19]. In contrast we took the values from all the standards melting curves (0%, 12.5%, 25%, 50%, 75%, 100% methylation standards) and derived an interpolation curve by the method of interpolating polynomials. Having obtained the interpolation curve for an experiment, we inputted the fluorescence of each sample to obtain a precise percentage of methylation of the template of interest. Both the present protocol and the method devised by Newman et al. [19] seem to be useful approaches to obtain quantitative (single) methylation values from MS-HRM.

**Figure 6:**
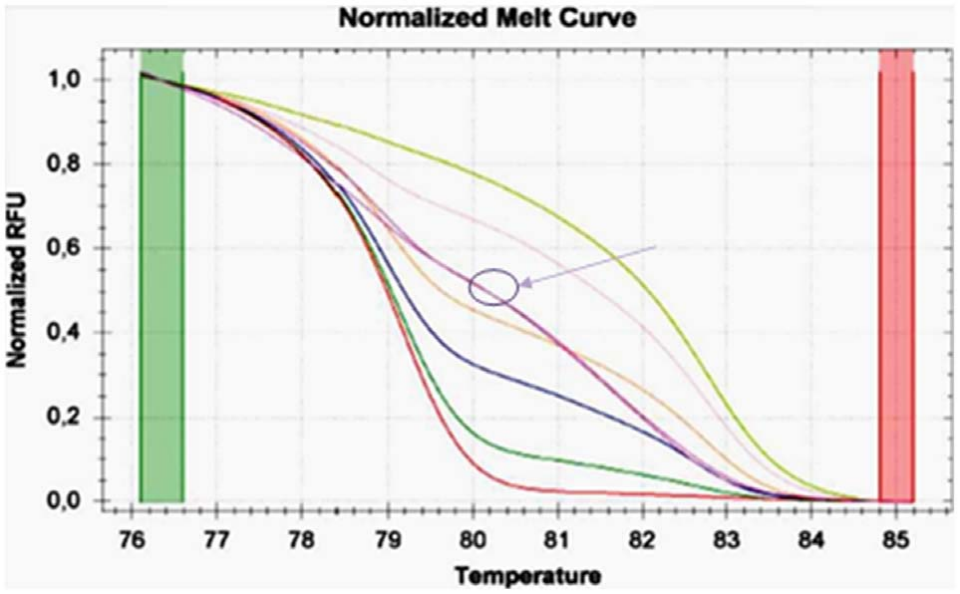
Heterogeneous methylation of *CDKN2A* gene in MS-HRM. The standards and a sample P25T in duplicate (highlighted). The arrow shows the unusual melting curve.

Some samples showed an unusual melting profile (Figure 6) and the corresponding estimates of methylation obtained by pyrosequencing showed quite different levels of methylation at each CpG site. This heterogeneous methylation across CpG sites within the domain of interest could explain the complex melting profile of some samples and further work could be required to examine such relationships with a wider range of samples. Candiloro and coworkers suggested that the characteristic profile of the melting curve, in samples showing heterogeneous methylation across the analyzed domain, could be used to identify those amplification products that require further investigation [20]. Quiellen et al. compared five different methylation techniques performed in five different laboratories around the world. The authors tested 13 different samples with theoretical percentage of methylation from 0% to 100% in a single run. The samples with the lowest proportion (2.5%) of methyation were identified as being methylated with both MSP (Methylation Specific PCR) and MS-HRM, whilst pyrosequencing and Methy-Light assays returned values of 4% and 5% respectively. It seems that all of these methods are sufficiently sensitive to detect relatively low percentages of methylation [21]. The comparison between the methods are thus consistent with our observations, although the authors give information about the sensitivity of MS-HRM only and do not attempt to calculate precise methylation percentages for each sample.

In conclusion, the outcomes from this study show that the novel approach which we have described can provide quantitative estimates of DNA methylation using the MS-HRM technique. Estimates of promoter methylation obtained for these two tumour suppressor genes (*APC* and *CDKN2A*) from both normal colorectal tissue and CRC tumour tissue ranged from zero to >80% and, across this whole range, MS-HRM gave estimates of methylation which were similar to those obtained by pyrosequencing. MS-HRM is a simple, closed tube, and relatively inexpensive method which has the potential to be a powerful tool for the quantification of methylation of specific CpG sites which may be valuable as biomarkers of prognosis and diagnosis in cancer. We also obtained evidence that the pattern of methylation across the domain of interest may affect the shape of the MS-HRM melt curve, with implications for methylation quantification. Six samples in *CDKN2A* methylation analysis showed heterogeneous methylation (unusual melting curve) by means of MS-HRM method; when we compared these results with those obtained by pyrosequencing, we observed a difference in methylation only in three samples, precisely the outliers obtained with the Bland-Altman plot (Figure 5A). The other three samples were similar in terms of methylation observed with both MS-HRM and pyrosequencing techniques. The main explanation of the outliers could be due to the primers design as described in our paper, where we referred to Wodjacz and coworkers guidelines for MS-HRM primer design [17]. In other words, in case of differential methylation of CpG sites included in the sequence recognized by MS-HRM primers, we can expect to have some differences in binding and amplification, depending on the ratio of methylated/unmethylated template. In case of heterogeneous methylation it is not yet clear if we can use MS-HRM technique to estimate a precise value of methylation using the calculation algorithm described in this paper, what is clear is that MS-HRM allows a rapid, cost-effective and easy detection of samples showing heterogenous methylation (Figure 6) that can be further processed by means of other available techniques in order to clarify the reasons of heterogeneity.
